# Case report: Coexistence of Jacobs syndrome, congenital adrenal hyperplasia, and ambiguous genitalia in a male infant

**DOI:** 10.1002/ccr3.8097

**Published:** 2023-11-09

**Authors:** Qaisar Ali Khan, Faiza Amatul‐Hadi, Amritpal Kooner, Amber Lee, Rahma Ahmed, Adithya Nadella, Harshawardhan Pande, Yaxel Levin‐Carrion, Muhammad Afzal, Moses Alfaro

**Affiliations:** ^1^ Khyber Teaching Hospital MTI KTH Peshawar Pakistan; ^2^ Mercer University School of Medicine Macon Georgia USA; ^3^ Chicago College of Osteopathic Medicine Downers Grove Illinois USA; ^4^ Arkansas College of Osteopathic Medicine Fort Smith Arkansas USA; ^5^ Kennesaw State University Kennesaw Georgia USA; ^6^ Nanjing Medical University Nanjing China; ^7^ Saint Louis University St. Louis Missouri USA; ^8^ Rutgers New Jersey Medical School Newark New Jersey USA; ^9^ St. George's University School of Medicine True Blue Grenada; ^10^ Long School of Medicine at the University of Texas Health Science Center San Antonio San Antonio Texas USA

**Keywords:** congenital adrenal hyperplasia, Jacobs syndrome, virilization, XY karyotype

## Abstract

**Key Clinical Message:**

Jacobs syndrome and congenital adrenal hyperplasia are separate entities but share common clinical features such as ambiguous genitalia. Further studies are needed to conclude the relationship between Jacobs syndrome and congenital adrenal hyperplasia.

**Abstract:**

A 5‐month‐old male infant was evaluated for ambiguous genitalia. Examination revealed cryptorchidism, inguinal hernia, long phallus, and Grade 3 scrotal hypospadias. Serum 17‐OH progesterone was high and chromosomal analysis showed 47XYY/45XO. A diagnosis of Jacobs and CAH was made. The parents were counseled about the patient's condition. He was given hydrocortisone and referred to the pediatric surgeon for further management.

## 1 INTRODUCTION

Congenital adrenal hyperplasia (CAH) describes a family of autosomal recessive diseases caused by gene mutations encoding enzymes in the cortisol biosynthesis pathway. The clinical and biochemical manifestations of CAH are quite variable. The most common form of CAH, making up more than 95% of congenital adrenal hyperplasia cases, results from 21‐hydroxylase deficiency (21OHD), due to loss of function mutation in CYP21A2. Classic CAH from 21OHD occurs in 1:10,000 to 1:20,000 live births with a female/male ratio of 2:1.^1^ Disease severity and phenotypic presentation vary depending on the location and extent of gene mutations or deletions, which lead to complex allelic variations.

Jacobs syndrome also known as 47, XYY syndrome, is caused by the insertion of a male Y chromosome to 46, XY. It occurs in 0.1% of the male population. Additionally, there are no specific clinical manifestations in most boys with the XYY karyotype. Diagnosis of an XYY karyotype is delayed (mean age at diagnosis is 17.1 years) and only 15% of patients are diagnosed with XYY syndrome. The karyotype 47, XYY is relatively common, but its phenotypes are not well understood. Patients may vary greatly, ranging from no phenotype and relatively few abnormalities to multi‐systemic symptoms; for a specific symptom, the severity can vary among individuals.^2^

XYY has also been sporadically reported in connection with several cases of disorder of sex development (DSD). Ambiguous genitalia is the condition commonly found in disorders of sex development (DSDs) characterized by imperfect differentiation of external genitalia between males and females. Sex Chromosome mosaicisms like 45 X0/46, XY, or 45 X/47 XYY have been considered major causes of ambiguous genitalia. Such DSD phenotypes of XYY patients include micropenis, testicular dysplasia, true‐hermaphrodite, and complete sex reversal.^3^ Congenital adrenal hyperplasia due to 21‐hydroxylase deficiency is a common cause of ambiguous genitalia in genotypically normal female infants (46XX). Most males have no signs of CAH at birth. However, some may present with hyperpigmentation and penile enlargement while those with salt‐wasting disease present early with hyponatremia and hypovolemia. Males with non‐salt‐wasting disease present later with signs of virilization. In rare forms, males are under‐masculinized.^4^ This report highlights the rare case of congenital adrenal hyperplasia coexisting with Jacobs syndrome presenting as ambiguous genitalia.

## 2 CASE PRESENTATION

A 5‐month‐old infant was brought to the outpatient department of a tertiary care hospital for ambiguous genitalia. The infant was born at 36 weeks of gestation to a 26‐year‐old primigravida mother. The marriage was non‐consanguineous and the paternal and maternal ages were 30 and 26 years respectively. The pregnancy was complicated due to delayed fertility for 3 years and the mother revealed the use of herbal medicines during this period. The infant had a birth weight of 3 kg with an APGAR score of 9 at both 1 and 5 min.

**FIGURE 1:**
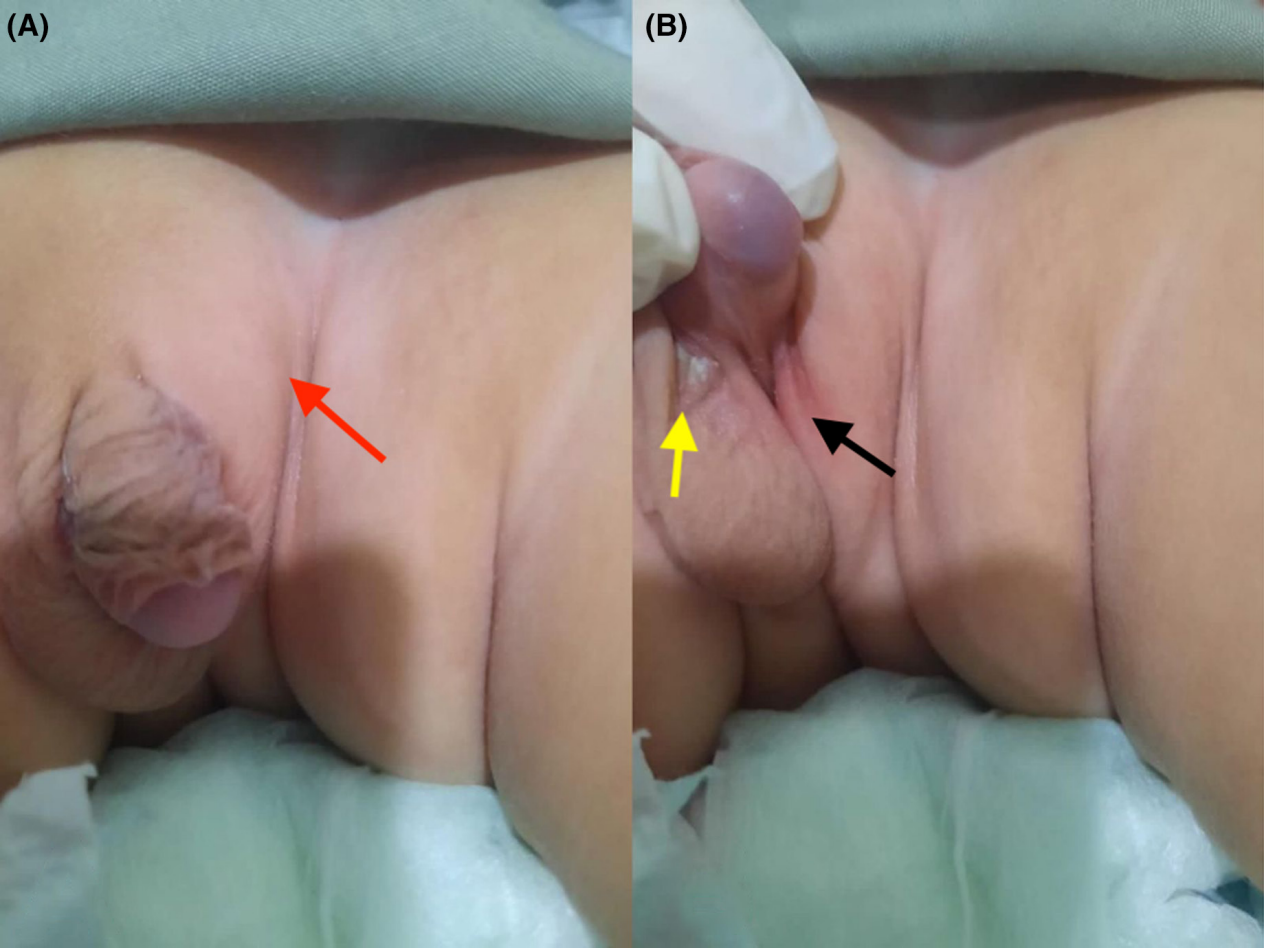
(A) Showing a long phallus and inguinal swelling represented by the red arrow, (B) left‐sided empty scrotum, and scrotal hypospadias as represented by black and yellow arrows respectively.

The infant had a height of 90 percentile, a weight of 50 percentile on the CDC chart, and attained age‐appropriate milestones. On examination of the genitalia, there were left‐sided undescended testes, a long phallus with a stretched penile length of length of 5.3 cm, and Grade 3 scrotal hypospadias. Left‐sided inguinal swelling was also noted on clinical examination as shown in Figure 1.

**FIGURE 2:**
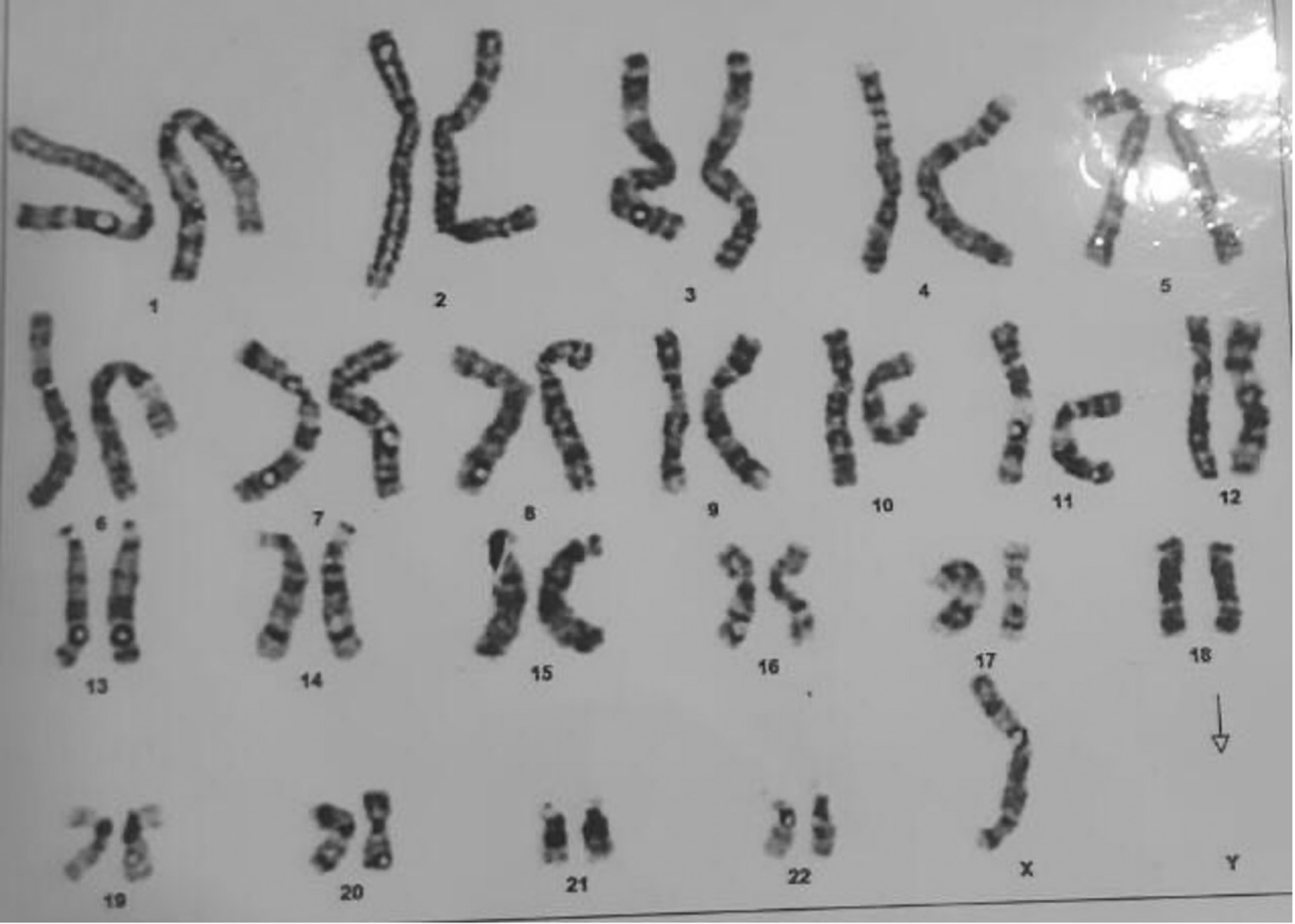
Chromosomal analysis showing 45X0.

Ultrasound of the abdomen and pelvis was ordered that showed a left‐sided undescended testis with a left‐sided inguinal hernia. Laboratory investigations including hormonal levels were requested that revealed increased serum 17‐OH progesterone levels as shown in Table 1. A diagnosis of congenital adrenal hyperplasia was made and further genetic study was performed to explore the potential genetic abnormalities associated with the observed features. A total of 30 cells were selected for chromosomal analysis and 21 showed 45X0 while the other 9 showed 47XYY in a mosaic manner that confirmed the diagnosis of Jacobs syndrome as shown in Figure.2.

**TABLE 1:** Laboratory investigations.

Investigation	Results	Normal range
Serum cortisol (9 am)	2.6 nmol/L	4.4–25
Serum testosterone	4.9 nmol/L	0.3–4.9
17‐OH progesterone	28 nmol/L	12–20
Serum sodium	138 mEq/L	135–145
Serum potassium	3.8 mmol/L	3.5–5.0
Serum chloride	98 nmol/L	90–110

The parents were counseled about the condition and its long‐term implications, such as the need for speech and occupational therapy and behavioral interventions in the future. The patient was started on hydrocortisone 10 mg/m^2^/day in two divided doses for 4 weeks and pediatric surgical and urological consultation was arranged to address the patient's urogenital defects. Frequent follow‐up was suggested to regularly monitor the patient's hormones, growth, and development.

## 3 DISCUSSION

Congenital adrenal hyperplasia (CAH) is a group of inherited disorders that are present at birth where the adrenal glands are hyperplastic, most commonly resulting from mutations or deletions of CYP21A. In CAH, the body is missing an enzyme that stimulates the adrenal gland to release cortisol. Disease severity and phenotypic presentation vary depending on the location and extent of gene mutations or deletions, which lead to complex allelic variations. Almost 300 CYP21A2 mutations have been identified, making genotyping these individuals a cumbersome undertaking. CAH can be seen as a continuum from salt wasting to mild forms but is divided into two categories for convenience: classical approximately 67% (“salt‐losing,” severe, ex‐congenital), and nonclassical approximately 33% (“non‐salt‐losing” or “simple‐virilizing,” less severe, formerly known as late‐onset or cryptic) according to the degree of aldosterone deficiency Patients with classic CAH may present as simple virilizing CAH or salt‐wasting CAH and are usually diagnosed in infancy while patients with non‐classical CAH may be asymptomatic or present with a milder form of virilization postnatally.^5^ CYP21, found on chromosome 6p, near the human leukocyte antigen gene cluster, is the gene for adrenal 21‐hydroxylase. Specific mutations may be linked to a degree of enzymatic dysfunction and the clinical manifestation of 21‐hydroxylase insufficiency. Minor mutations on both alleles of the 21‐hydroxylase gene are found in patients with non‐classic forms^6^ A retrospective cohort study conducted by Gidlöf et al. in Sweden found the CYP21A2 genotype in 81% of the patients, reflecting improved diagnostic usage of genetic studies.^7^ The infant we are reporting was diagnosed solely based on clinical findings and laboratory values. Due to financial constraints and resource availability, genetic studies could not be conducted in our case.

It is shown that up to 29.3% of CAH patients had adrenal tumors. Abdominal ultrasound is the modality of choice for small‐sized pediatric patients due to the lack of ionizing radiation, lower cost than cross‐sectional imaging, and extensive availability.^8^ In our situation, the ultrasound of the infant resulted in the adrenal glands being normal, ruling out the likelihood of an adrenal tumor.

Symptoms of Jacobs syndrome may be quite vague during childhood, and for this reason, most children go undiagnosed. However, Men with Jacobs syndrome who do display symptoms are most likely to exhibit tall stature and macrocephaly. Developmental delays and behavioral issues have been noted, as well as atonia, clinodactyly (medial curvature of a digit, i.e., fifth finger toward the fourth), and hypertelorism. The incidence of asthma and autism spectrum disorder also appears to be increased in these individuals.^9^ Other conditions such as Marfan syndrome and Sotos syndrome should be ruled out, In contrast to Jacobs syndrome, Marfan syndrome often presents with cardiac abnormalities such as aortic root dilatation and mitral valve prolapse. Sotos syndrome, also known as cerebral gigantism, is a rare genetic condition caused by a mutation in the NSD1 gene. Hallmark features include excessive growth during childhood, macrocephaly, learning disabilities, hypotonia, and seizure disorders.^10^ Our patient had none of them.

Patients with Jacobs syndrome have been found to have an increased incidence of certain diseases. These include asthma, seizure disorders, and tremors. Some 47, XYY patients have been noted to have genitourinary abnormalities such as microphallus, hypoplastic scrotum, cryptorchidism, and hypospadias. These patients are also at an increased risk for learning disabilities, ADHD, autism spectrum disorder, and speech difficulties.^11^

The diagnosis of both CAH and Jacobs syndrome can be done prenatally with amniocentesis or chorionic villus sampling, and treatment involves dexamethasone administered at or before 10 weeks of gestation. A study conducted by Carlson et al. found that prenatal diagnosis and therapy of 21‐hydroxylase deficiency is safe and effective in lowering or eliminating virilization in the affected female, sparing the newborn female the repercussions of genital ambiguity, sex misassignment, and gender confusion.^12^ However, in our case, the mother did not undergo routine antenatal visits during the pregnancy leading to the failure of antenatal diagnosis of CAH as well as Jacobs syndrome.

There is no treatment for XYY syndrome. The treatment option is generally only supportive, with attention given to the comorbidities of the patient. While medication cannot treat XYY syndrome, some medications can be used to treat conditions related to the syndrome. A person with XYY syndrome can get help with any learning or developmental delays through speech therapy, occupational therapy, or other assistance. Therapy can also help with ADD/ADHD, social interactions, and other behavioral problems. The treatment for CAH is based on replacing normal glucocorticoid and mineralocorticoid needs, as well as psychological support^2^, ^13^ The patient is currently receiving hydrocortisone. The parents of the infant were counseled about the need for genital surgery and psychological support.

## 4 CONCLUSION

This rare case highlights the co‐existence of CAH and Jacobs syndrome (47, XYY Syndrome) that leads to ambiguous genitalia and various urogenital abnormalities and underscores a stepwise diagnostic approach in patients with atypical presentation. A multidisciplinary approach including endocrinology, surgery, and genetics is necessary for the proper management of complexities associated with the condition. Further research is needed to find the association between CAH and Jacobs syndrome in patients with ambiguous genitalia.

## AUTHOR CONTRIBUTIONS

**Qaisar Ali Khan:** Conceptualization; data curation; investigation; writing – review and editing. **Faiza Amatul‐Hadi:** Writing – review and editing. **Amritpal Kooner:** Writing – original draft. **Amber Lee:** Writing – original draft. **Rahma Ahmed:** Writing – original draft. **Adithya Nadella:** Writing – original draft. **Harshawardhan Pande:** Writing – original draft. **Yaxel Levin‐Carrion:** Writing – original draft. **Muhammad Afzal:** Writing – original draft. **Moses Alfaro:** Writing – original draft.

## FUNDING INFORMATION

No funding sources were utilized in the composition of the research.

## CONFLICT OF INTEREST STATEMENT

The authors report no conflict of interest.

## CONSENT

A written informed consent was taken from the infant's parents to publish this case or any accompanying images.

## Data Availability

The data that support the findings of this study are available from the corresponding author upon reasonable request.
